# The Role of microRNA in the Inflammatory Response of Wound Healing

**DOI:** 10.3389/fimmu.2022.852419

**Published:** 2022-03-21

**Authors:** Yuanyuan Jiang, Xiang Xu, Long Xiao, Lihong Wang, Sheng Qiang

**Affiliations:** Center Laboratory, Zhangjiagang TCM Hospital Affiliated to Nanjing University of Chinese Medicine, Zhangjiagang, China

**Keywords:** microRNA, skin, wound healing, inflammation, exosomes

## Abstract

Wound healing, a highly complex pathophysiological response to injury, includes four overlapping phases of hemostasis, inflammation, proliferation, and remodeling. Initiation and resolution of the inflammatory response are the primary requirements for wound healing, and are also key events that determines wound quality and healing time. Currently, the number of patients with persistent chronic wounds has generally increased, which imposes health and economic burden on patients and society. Recent studies have found that microRNA(miRNA) plays an essential role in the inflammation involved in wound healing and may provide a new therapeutic direction for wound treatment. Therefore, this review focused on the role and significance of miRNA in the inflammation phase of wound healing.

## 1 Introduction

Our skin is a highly adaptable and multifunctional organ that can heal quickly and efficiently after being exposed to the harsh external environments that can include physical or chemical irritants and ultraviolet radiation. The structure of the skin is divided into three layers: the epidermis, which is mainly composed of microlayers differentiated from keratinocyte cells; the dermis, composed of two connective tissue layers rich in collagens (I, III type), elastin, fibroblasts, macrophages, lymphocytes, sensory neurons, and blood vessels; the hypodermis, which is the layer of adipose tissue (1). Efficient and coordinated function between the three layers of skin structures is required to restore the damaged barrier after injury. Although the skin has a strong regenerative capacity, its repair ability can be weakened due to individual physiological factors such as advanced age, diabetes or obesity. This may lead to wounds that take longer than 12 weeks to heal (chronic wounds). It has been reported that millions of individuals suffer from skin injuries every year and are often accompanied by infections that are difficult to heal (2). Therefore, it is extremely urgent to find effective clinical treatment methods for wound healing (3).

The wound healing process is characterized by four sequential but overlapping classical phases: hemostasis, inflammation, proliferation/migration, and remodeling (4). After skin injury, a large number of inflammatory cells are recruited to the wound site to participate in the repair process and resisting pathogen infection (5). In addition, some intracellular inflammatory signaling pathways and inflammatory mediators are involved in regulating the initiation and dispersal of inflammation (6). However, chronic wounds often manifest as severe dysregulation of inflammation and continuous destruction of tissues (7). The inflammatory response is the crucial to successful wound healing, and an imbalance between pro-inflammatory and anti-inflammatory signals may lead to the occurrence of infection, failure to heal, and the formation of hypertrophic scars and keloids (8, 9).

In recent years, several epigenetic phenomena have been found to be involved in regulating gene expression and coordinating various biological processes that drive tissue repair (10). For example, non-coding gene microRNA (miRNA) regulates gene expression by inhibiting the translation process, which is different than the traditional regulation of gene expression through transcription. Recently, the important role of miRNA in wound healing has been widely reported (11). In this review, we mainly reviewed the evidence that miRNA was involved in regulating the inflammation stage of wound healing. We also discussed current miRNA-based treatment strategies and the future direction of using miRNA as a target for wound treatment.

## 2 Inflammatory Phase of Wound Healing

Inflammation is the primary defense against pathogen invasion at the wound site and usually begins in the first few hours after tissue injury (12). The injury leads to the activation of the coagulation system, which successfully stops bleeding and provides the necessary matrix for the initiation of the inflammatory response. Tissue-resident cell surface pattern recognition receptors (PRRs) are recognized by Damage Associated Molecular Patterns (DAMPs) or Pathogen Associated Molecular Patterns (PAMPs), which in turn release specific chemokines and pro-inflammatory cytokines to recruit inflammatory cells (13). At the same time, the intracellular inflammatory signaling pathways, such as toll-like receptors (TLRs) and nuclear factor kappa B (NFκB) at the wound site are activated to trigger a signaling cascade (14).

**Figure 1:**
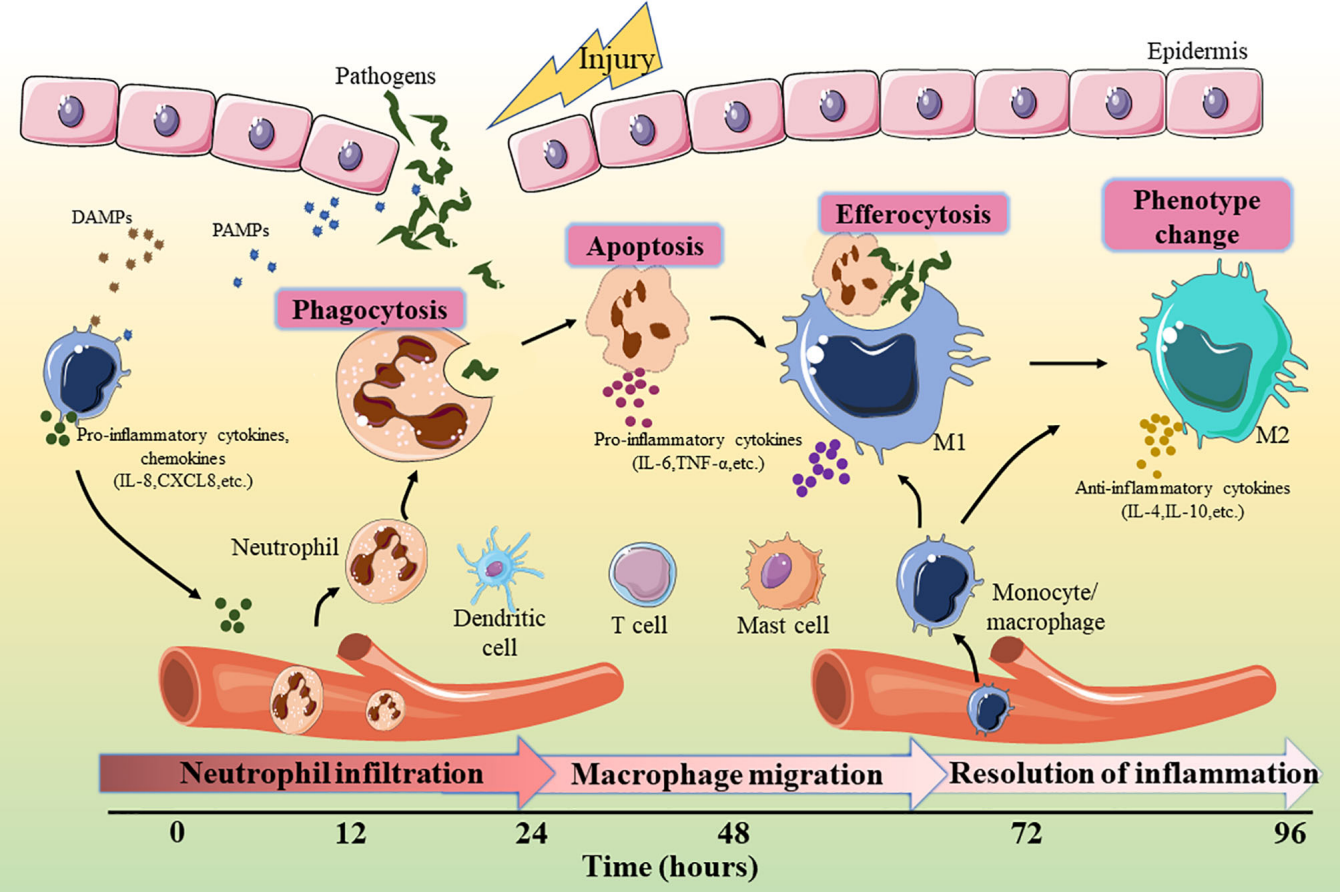
Key events involved in the inflammatory phase of wound healing. DAMP, damage-associated molecular patterns; PAMP, pathogen-associated molecular patterns; IL, interleukin; TNF, tumor necrosis factor.

As shown in **Figure 1**, the inflammatory cells recruited in the early stage of injury are mainly neutrophils, which are the main defense against bacteria (15). One day after injury, the number of neutrophils accounts for approximately half of all cells at the wound site and (16). Neutrophils, derived from bone marrow, are usually not observed in intact skin. There are a large number of receptors on the surface of these cells that detect the attraction signals of the chemo-attractants released from tissue-resident macrophages (17). Subsequently, neutrophils eliminate necrotic cells and pathogens through phagocytosis, the formation of extracellular traps, and antimicrobial mechanisms such as the release of high concentrations of reactive oxygen species (ROS) and nitric oxide (NO) (18–20). ROS such as hydrogen peroxide (H_2_O_2_) exert anti-microbial activities by destroying lipids, proteins or DNA, and their production depends on the function of the nicotinamide adenine dinucleotide phosphate (NADPH) oxidase. Phagocytosis of neutrophils with ROS generation and degranulation cooperate to defense and kill pathogens (21). Within 2-4 days after injury, circulating monocytes are recruited to the wound site, where they differentiate into different macrophage subsets according to local microenvironment (22). Macrophages are the crucial effector cells in wound healing and have variety of phenotypes and versatility (23). They differentiate into the pro-inflammatory type (M1 macrophages) in the early stage of inflammation (24). These macrophages release a variety of inflammatory cytokines (such as interleukin (IL)-6, IL-1 and tumor necrosis factor (TNF)-α) to play a pro-inflammatory roles (25). In addition, M1 macrophages are involved in the phagocytosis and clearance of some pathogens and apoptotic neutrophils (26). During the later period of inflammation, anti-inflammatory macrophages (M2 macrophages) are generated through new differentiation of recruited monocytes or phenotypic transitions of previous pro-inflammatory macrophages (27). These macrophages can release anti-inflammatory cytokines, such as IL-4, IL-10, and IL-13, to promote inflammation resolution by reducing ROS production and neutrophil infiltration. At the same time, growth factors such as vascular endothelial growth factor (VEGF) are also released to initiate tissue repair (28). Although the effects of neutrophils and macrophages are irreplicable, it has been reported that other myeloid cells, including mast cells, dendritic cells (DC), and T cells, are also critical in the inflammatory phase of wound healing (29–31).

## 3 miRNA Biogenesis and Physiology

miRNA is currently the most widely studied non-coding RNA (ncRNA) with a length of about 18-25 nucleotides (32). It plays a regulatory role by combining with the 3’ untranslated region (UTR) of a specific messenger RNA (mRNA) to silence gene expression (33). The single-stranded structure of miRNA determines the complexity of its function. A signal miRNA can participate in blocking the translation of multiple mRNAs, while the translation inhibition of some mRNAs requires the synergy of multiple miRNAs (34, 35). It has been reported that the translation of more than 60% of human coding genes is regulated by miRNAs, so they play important roles not only in maintaining normal physiological processes, but also in the occurrence or resolution of a variety of diseases (36, 37).

**Figure 2:**
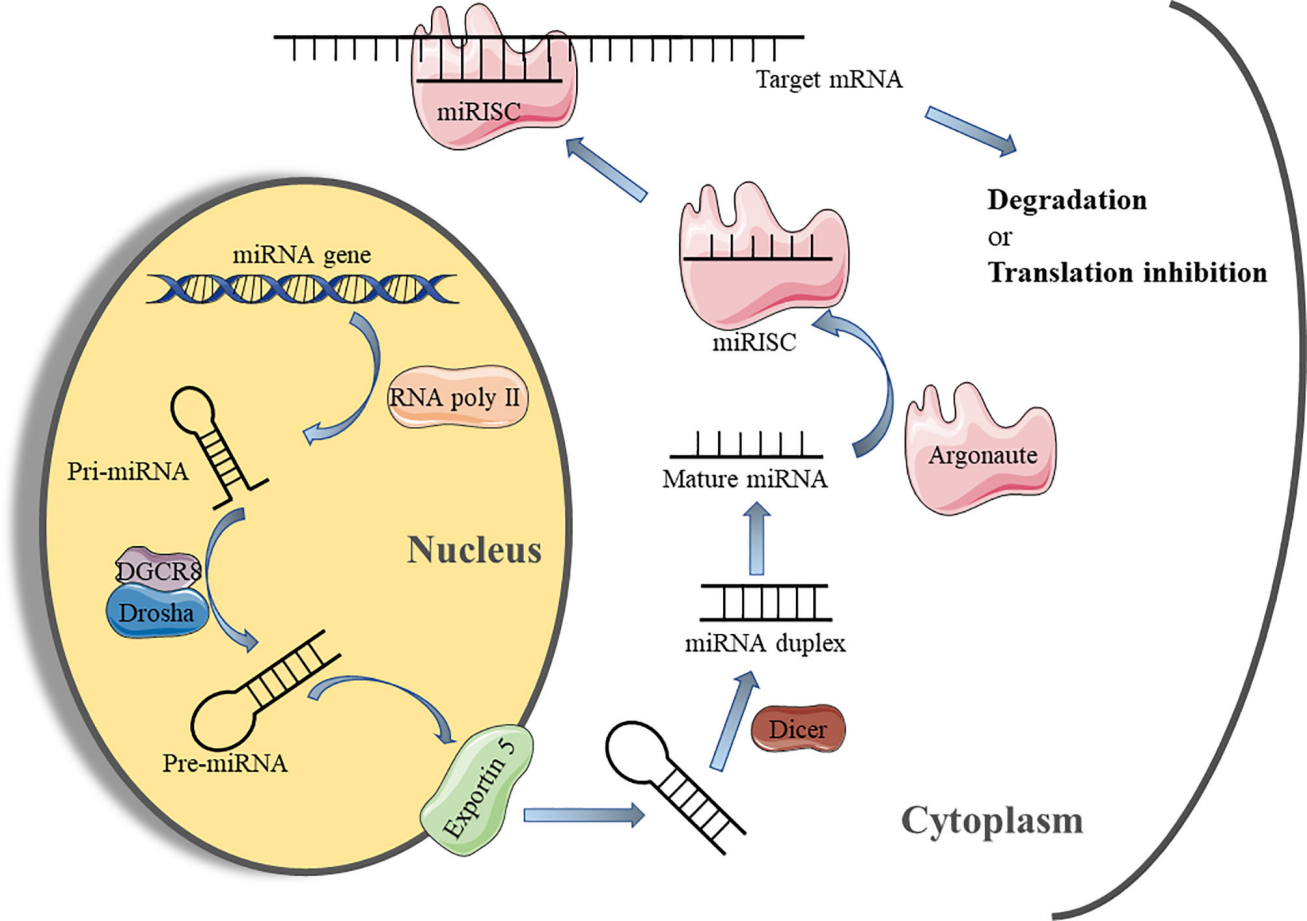
miRNA biosynthesis process and mechanism of action. miRNA, micro-RNA; Pri-miRNA, primary miRNA; Pre-miRNA, precursor miRNA; DGCR8, DiGeorge syndrome critical region gene 8; miRISC, microRNA-induced silencing complex.

The maturation of miRNAs requires a multi-step biological process (**Figure 2**). Initially, the miRNA is transcribed under the mediation of RNA polymerase II in the nucleus to form the long stem-loop structure, which is called primary-miRNA (pri-miRNA). Subsequently, pri-miRNA is cleaved into 60-70 nucleotide precursor-miRNA (pre-miRNA) by RNase III enzyme, Drosha, and its cofactor DiGeorge syndrome critical region gene 8 (DGCR8), which is then exported to the cytoplasm *via* the Exportin 5 transporter (38). In the cytoplasm, pre-miRNA is processed by another RNase III enzyme, Dicer, to generate miRNA duplexes. Finally, the miRNA duplex is dissociated into an active “guide strand” which forms a miRNA-induced silencing complex (miRISC) with the assistance of the argonaute protein family and a “passenger strand” that may be degraded (39). The miRNA in the RISC matches the 3’UTR region of the target mRNA through its own “seed region” (nucleotides 2-8 at 5’ end) for complementary pairing. This combination leads to the degradation of mRNA or inhibition of protein synthesis to regulate target gene expression (40).

miRNAs not only exist as critical epigenetic regulators, but their expression is also highly modulated by epigenetic mechanisms, including post-translational modifications (PTMs) of histones and DNA methylation (41). miRNA expression can be increased or decreased through different epigenetic modifications. Besides, specific RNA-binding proteins (RBPs) can modulate miRNA biogenesis by recognizing pre-miRNA sequences (42). Similarly, the inactivation of specific components in miRNA biogenesis can also affect miRNA production. For example, a recent study found that ubiquitination of DDX17 (a cofactor in miRNA biogenesis) effectively reduced miRNA expression (43). In short, the regulation mechanism of miRNA expression is strictly controlled.

Although the classical pathway of miRNA biogenesis and function have been well studied, this is not sufficient for the complexity of miRNA-mRNA interactions. Researchers find that miRNA biogenesis can also be accomplished through Drosha/DCGR8 or Dicer independent pathways (34). Moreover, non-canonical binding events of multiple miRNA-mRNA interactions have also been reported, such as pairing with mRNA 5’UTR or coding region (44, 45). Initially, miRNAs were thought to form miRISC mediated post-transcriptional regulation mainly in the cytoplasm. However, it was proved that some mature miRNAs can be detected in the nucleus and induce the degradation of nuclear mRNAs (46). Besides most repression of target gene expression, there are some studies also demonstrate that certain miRNAs expression can induce transcriptional activation of target genes (34). The specific mechanisms by which miRNAs mediate the regulation of gene transcription or translation through these non-canonical means are unclear, but this may provide new explanations for the complex regulation of miRNAs on a variety of biological processes.

## 4 miRNA in Wound Inflammation

Emerging evidence suggests that miRNA plays a key role in regulating wound inflammation (47, 48). Interestingly, it has been shown that the development and function of various immune cell lineages are modulated by miRNA (49, 50). In fact, miRNAs have a great potential in regulating both induction and resolution of inflammatory response (50, 51). miRNA can maintain tissue homeostasis at the wound site to promote healing by regulating the differentiation and development of immune cells, controlling the activation of inflammatory signaling pathways, and producing inflammatory mediators (**Table 1**).

**Table 1:** miRNAs involved in the inflammatory process of wound healing.

miRNA	Target	Process	Effect on wound healing	Reference
**Immune cell**				
** miR-142-3p/5p**	Small GTPase	Promote the migration of neutrophils	↑*	(52)
** miR-223**	Mef2c	(a) Inhibit the activation of neutrophils	**↑**	(53, 54)
		(b) Induce M2 macrophage polarization		
** miR-139-5p**	EIF4G2	Inhibits neutrophils differentiation	**↓**	(55)
** miR-21**	PTEN	(a) Enhance the endocytosis of macrophages	**↑**	(56–58)
		(b) Promote M1 macrophage polarization		
		(c) Promote DCs differentiation		
** miR-155**	SOCS1	(a) Increase inflammatory cell accumulation and activation	**↑**	(59–61)
		(b) Promote the differentiation of Th17 and Th9 cells		
**Inflammatory pathway**				
** miR-150-5p**		Activate TLRs signaling pathway	**↓**	(62)
** miR-21**	PDCD4	Restrict the TLR4 activity	**↑**	(63)
** miR-146a**	IRAK1/TRAF6	Inhibit TLR4 signaling pathway activation	**↑**	(64, 65)
** miR-132**	HB-EGF	Suppress the activation of NF-κB signaling	**↑**	(66)
** miR-223**	CUL1a/b, TRAF6, TAB1	Suppress the activation of NF-κB signaling	**↑**	(67)
** miR-34**	LGR	Enhance the activity of the NF-κB signaling pathway	**↓**	(68)
** miR-19a/b**	SHCBP1	Inhibit p65 nuclear translocation	**↑**	(69)
** miR-20a**	SEMA7A	Inhibit p65 nuclear translocation	**↑**	(69)
** miR-17**	STAT3	Inhibit JAK/STAT signaling pathway activation	**↓**	(70)
**Inflammatory mediator**				
** miR-203**	TNF-α, IL-24	Alleviate skin inflammation	**↑**	(71)
** miR-23b**		Decrease proinflammatory cytokines and increase anti-inflammatory cytokines	**↑**	(72)
** miR-149, miR-497**		Inhibit secretion of proinflammatory cytokines	**↑**	(73, 74)
** miR-16, miR-146a, miR-26a/b**	COX-2	Relieve inflammatory response	**↑**	(75, 76)
** miR-27b**	p66^shc^	Reduce ROS production	**↑**	(77)
** miR-375**	TIMP-1	Reduce ROS production	**↑**	(78)
** miR-5591-5p**	AGEs	Reduce ROS production	**↑**	(79)

*↑, promote wound healing; ↓, impede wound healing.

### 4.1 miRNAs and Immune Cells

#### 4.1.1 Neutrophils

Infiltration and activation of neutrophils are markers of early inflammatory response initiation in wound healing. *Staphylococcus aureus* is a common infectious bacterium that exhibits overgrowth in chronic skin wounds (such as diabetic wounds), and is associated with wound deterioration (80). Tanaka K et al. found that the healing speed of skin wounds infected by Staphylococcus aureus (*S. aureus*) in miR-142-deficient mice was significantly slower than that of wild-type mice (52). Further research revealed that the miR-142 family (miR-142-3p and miR-142-5p) promoted the migration of neutrophils by regulating the translation of small GTPase in neutrophils and enhanced the ability of the wound site to resist bacterial infection (52). miR-223, a miRNA primarily expressed in myeloid cells (especially neutrophils) is involved in regulating the development of various cancers, infection, and inflammation (81). It has been verified that miR-223 inhibited the differentiation of human myeloid cells by targeting Mef2c and was an important regulator of granulocyte formation (53). This study showed that the activity of neutrophils at the wound site was substantially increased in miR-223-deficient (miR‐223^Y/−^) mice compared with wild type (WT) mice (82). A recent study reported that Eukaryotic Translation Initiation Factor 4 Gamma 2 (Eif4g2) expression could be targeted by miR-139-5p, which limited the differentiation of neutrophils. After knocking down the expression of miR-139-5p in mice, neutrophil activation was increased and wound healing of *S. aureus* infection was improved (55).

#### 4.1.2 Macrophages

Macrophages differentiated from monocytes are essential in all wounds and are present for almost the entire inflammatory phase, whether it is the elimination of potential pathogens or the subsequent resolution of inflammation (83). The endocytosis function of macrophages is the key to effectively eliminate apoptotic or necrotic cells at the injury site. The expression of miR-21 is up-regulated after macrophages have successfully engulfed apoptotic cells (56). Furthermore, the enhanced expression of miR-21 in turn promotes the endocytosis of macrophages (56). Recently, Liechty C et al. found that miR-21 participated in regulating the polarization of macrophages and induced the production of pro-inflammatory macrophages (M1 type) in diabetic wounds (57). In contrast, miR-223 has been verified to effectively induce macrophages polarization into an anti-inflammatory M2 phenotype at the wound site (54). A previous study showed that miR-155 deficiency was beneficial in the promotion of wound healing (84). Subsequently, Ye J et al. found that the accumulation and activation of inflammatory cells such as neutrophils and macrophages in diabetic wounds was reduced after the addition of miR-155 inhibitors (59).

#### 4.1.3 Other Immune Cells

DCs are a special type of antigen-presenting cell and are important in the immune response process. It has been reported that miR-21 activated the protein kinase B/phosphatidylinositol3-kinase(AKT/PI3K) signaling pathway to promote DC differentiation by inhibiting the expression of phosphatase and tensin homologue (PTEN) in skin wounds (58). In addition, the differentiation of the T cells of the adaptive immune system is also regulated by miRNA. miR-155 has been confirmed to be involved in promoting the differentiation of Th17 and Th9 cells, which was achieved by inhibiting the expression of suppressor of cytokine signaling 1(SOCS1) (60, 61). All the above evidence indicates the importance of miRNA in regulating the differentiation and activation of immune cells, which are also the keys to the initiation and resolution of wound inflammation.

### 4.2 miRNAs and Inflammation-Related Signaling Pathway

#### 4.2.1 The TLR Pathway

TLRs, considered immune sensors, are involved in the formation of the first line of defense against invading pathogens and play a fundamental role in regulating inflammatory diseases (85). As transmembrane proteins, the structure of TLRs consists of a leucine-rich extracellular ligand binding domain, a transmembrane domain, and an intracellular Toll-IL-1 Receptor (TIR) signal domain. TLRs recognize PAMPs or DAMPs and induce signal cascade by recruiting adaptor molecules that interact with TIR domain including myeloid Myeloid differentiation primary response 88 (MyD88), TIR-domain containing adapter-inducing interferon-β (TRIF), TIR domain-containing adaptor protein (TIRAP), and TRIF-related adaptor molecule (TRAM) (86). Most TLRs signaling is MyD88-dependent or independent, which leads to the activation of nuclear transcription factors such as NF-kB, p38/mitogen-activated protein kinase(MAPK), and c-Jun N-terminal kinase/activator protein-1(JNK/AP-1), triggering inflammatory responses (87). In fact, TLR signaling pathways play integral role in regulating wound healing. The expression and activation of specific TLRs regulated by different conditions determine whether to promote or inhibit the process of wound healing (88). Studies have suggested that non-coding gene miRNA have directly or indirectly participated in regulating the TLR signaling pathway (89). Gene Ontology (GO) and KEGG pathways analysis showed that miR-150-5p may be involved in the activation of TLRs signaling pathways after traumatic injury occurs (62). A previous study has confirmed that TLR4 is one of the pivotal factors leading to persistent inflammation in diabetic wounds. Knocking out the expression of TLR4 is beneficial to alleviate the inflammatory response and promotes wound healing in diabetic mice (90). miR-21 has been shown in an earlier study to restrict the TLR4 activity by targeting programmed cell death 4 (PDCD4) (63). Furthermore, miR-146a negatively regulates the activation of TLR4 signaling pathway by targeting IL-1 receptor-associated kinase 1 (IRAK1) and TNF receptor-associated factor 6 (TRAF6) to suppress immune response (64). Moreover, Liu et al. suggested that miR-146a down-regulated TLR4 expression and reduced the inflammatory response of fibroblast-like synovial cells (65).

#### 4.2.2 The NF-kB Pathway

The NF-kB transcription factor family, consisting of five members (NF-κB1 (p105/p50), NF-κB2 (p100/p52), RelB, p65 (RelA), and C-rel) modulates the expression of key genes in many cellular biological processes (91). The activation mechanism of NFκB is divided into classical and non-classical pathways. The classical pathway primarily promotes transcription by inducing the translocation of a free p65/p50 dimer into the nucleus to bind to the κB element on the target gene (92). It has been recognized that the regulation of the inflammatory process is one of the most important functions of classical NF-kB signaling pathway (93). Therefore, the NF-κB signaling pathway is closely related to wound inflammation and has complex crosstalk with miRNAs. Epidermal growth factor (EGF) is an important molecule that mediates the activation of NFκB signal (94). In keratinocytes, miR-132 inhibits NFκB activity by directly targeting silencing heparin-binding epidermal growth factor (HB-EGF) and reducing inflammation (66). Similarly, Zhou W et al. found that miR-223 could directly target multiple components (cullin-1 (CUL1) a/b, TRAF6, and transforming growth factor β(TGF-β)-activated protein kinase 1(TAB1))of the signal cascade to inhibit the activation of the classical NFκB pathway, which was conducive to the resolution of neutrophil inflammation (67). On the contrary, Wu J et al. demonstrated that miR-34 enhanced the activity of the NF-κB signaling pathway to regulate the inflammatory response of keratinocytes, which was mainly achieved by directly targeting the inhibition of leucine-rich repeat containing G protein receptor(LGR) gene expression (68). The latest research showed that miR-19a/b and miR-20a suppress p65 nuclear translocation by targeting SHC SH2 domain-binding protein 1(SHCBP1) and semaphorin 7A(SEMA7A), respectively, and they regulate TLR3-mediated activation of NF-κB signaling pathway (69). miR-31 has been reported to be highly expressed in human wound edge keratinocytes. The results of *in vitro* experiments demonstrated that miR-31 promoted the proliferation and migration of keratinocytes, which was beneficial to wound healing (95). Subsequently, Shi J et al. found that miR-31 was up-regulated by the NFκB signaling pathway during the inflammation phase to mediate the transition from wound inflammation to the remodeling phase (96). The above findings indicate that the complex regulatory network between miRNA and NFκB signaling plays an important role in in the initiation and resolution of wound inflammation.

#### 4.2.3 The JAK/STAT Pathway

**Figure 3:**
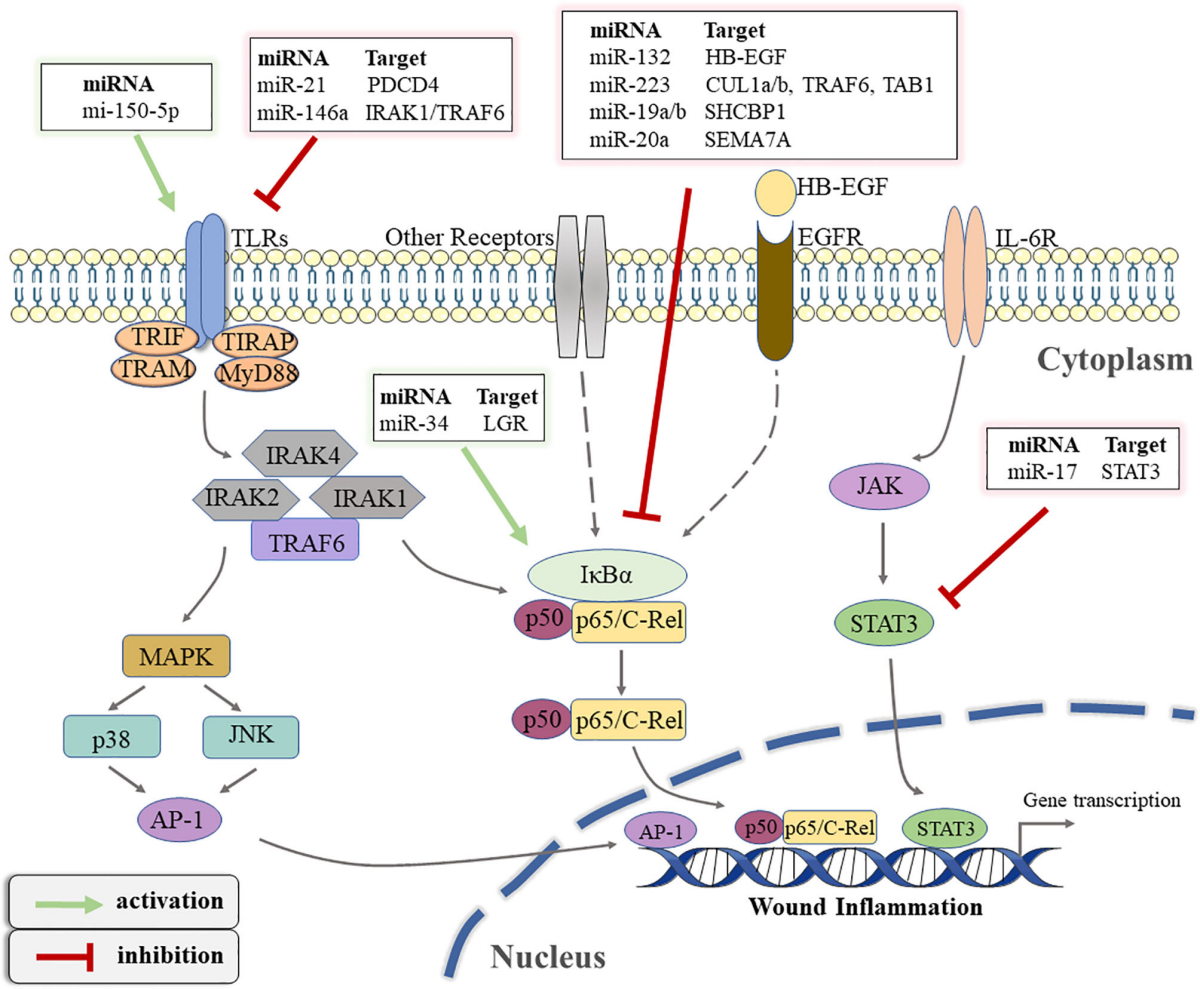
The schematic overview of miRNA-regulated inflammatory signaling pathways that occur during wound healing. TLRs, toll-like receptors; TIR, toll-IL-1 receptor; TRIF, TIR-domain-containing adapter-inducing interferon-β; TRAM, TRIF-related adaptor molecule; TIRAP, TIR domain-containing adaptor protein; MyD88, Myeloid differentiation primary response 88; IRAK, interleukin(IL)-1 receptor associated kinase; TRAF, tumor necrosis factor receptor-associated factor; MAPK, mitogen-activated protein kinase; JNK, c-Jun N-terminal kinase; AP-1, activator protein-1; PDCD4, programmed cell death 4; IκBα, inhibitor of kappa B alpha; LGR, leucine-rich repeat containing G protein receptor; EGF, epidermal growth factor; HB-EGF, heparin-binding epidermal growth factor; EGFR, EGF-receptor; CUL1, cullin-1; TAB1, transforming growth factor β(TGF-β)-activated protein kinase 1; SHCBP1, SHC SH2 Domain-Binding Protein 1; SEMA7A, semaphorin 7A; IL-6R, IL-6 receptor; JAK, Janus kinase; STAT3, signal transducer and activator of transcription 3.

In addition to the above signal pathways, the Janus kinase/signal transducer and activator of transcription(JAK/STAT) pathway is also involved in the regulation of wound inflammation (97). This signaling pathway involves the IL-6 receptor (gp130) family, which is associated with the initiation of immune responses (98). Based on its important role in a variety of inflammatory diseases, the STAT signaling pathway has been reported to be a target for the treatment of inflammation (99). miRNA has also been reported to regulate the JAK/STAT signaling pathway in wound repair. For example, Yang Z G et al. demonstrated that STAT3 expression was down-regulated in the miR-17 transgenic mice (70). Subsequently, a luciferase assay confirmed that miR-17 could directly target the 3′UTR of STAT3, which suppressed the activation of the JAK/STAT signaling pathway (70). **Figure 3** illustrates how various miRNAs target inflammatory pathway-related genes to participate in the regulation of wound inflammation. Further studies are required regarding the regulation of miRNA on some inflammation-related signaling pathways in the inflammatory phase of wound healing. This would provide new directions for accelerating wound healing and even the treatment of chronic refractory wounds.

### 4.3 miRNA and Inflammatory Mediators

Cytokines are recognized as “inflammatory mediators” secreted by immune cells and non-immune cells and are closely related to the inflammatory response (100). miRNA also plays an important role in regulating functional cytokine networks (101). A previous study showed that miR-203 directly targeted TNF-α and IL24 expression and alleviated skin inflammation (71). The expression of pro-inflammatory cytokines (such as TNF-α, IL-1β, and IL-6) in wound tissues of mice overexpressing miR-23b was significantly reduced, while the anti-inflammatory factor IL-10 was increased (72). In addition, miR-149 and miR-497 have also been found in recent studies to inhibit the secretion of pro-inflammatory cytokines and exert an anti-inflammatory effect in wound healing (73, 74). In addition to the cytokines secreted by cells, there are also some small molecular lipids derived from fatty acid metabolism that are also labeled as “inflammatory mediators” (102). Lipid mediators produced by free arachidonic acid metabolism, such as prostaglandins (PG), prostacyclin (PC) and thromboxanes (TX), are important chemicals that trigger and amplify wound inflammation (103). Some lipid mediators derived from polyunsaturated fatty acids (PUFAs) have been reported to be involved in the resolution of inflammation (102). In fact, cyclooxygenases (mainly COX1 and COX2) are key enzymes that catalyze the production of these lipid mediators (102). COX-2 has is a target of miR-16 and miR-146a to regulate inflammatory response (75). Kwon Y et al. found that miR-26a and miR-26b relieved allergic inflammation by inhibiting the expression of COX2 and forming a negative feedback loop (76).

ROS, also an essential mediator in regulating inflammatory response, play a significant role in wound healing and tissue repair. However, excessive ROS leads to oxidative stress and persistent chronic inflammation impairing tissue regeneration. Wang et al. observed that miR-27b overexpression accelerated wound healing in diabetic mice, partly by reducing mitochondrial ROS production through targeted adaptor protein p66^shc^ (77). In addition, miR-375 and miR-5591-5p were also shown to be involved in the inhibition of intracellular ROS production during wound healing by targeting metalloproteinases 1 (TIMP-1) and advanced glycation end products (AGEs), respectively (78, 79).

## 5 Exosomal miRNAs in Wound Inflammation

Exosomes, secreted by a variety of cells, are crucial components of intercellular information transmission (104). Many experiments have shown that exosomes were closely associated with maintaining skin homeostasis, regulating wound inflammation and promoting repair (16). For example, human mesenchymal stem cells (MSC) release exosomes through the paracrine mode to regulate the inflammatory microenvironment of the wound site and accelerate wound healing. These exosomal cargoes often contain some miRNAs, such as miR-21, miR-146a, and miR-181, which are carried to the wound site to participate in the resolution of inflammation (105). Scientists found that EVs isolated after adding miR-223 inhibitor to bone marrow MSC (BMMSC) significantly reduced the polarization of macrophages to M2 type and delayed cutaneous wound healing (106). This indicates that BMMSC transferred miR-223 to the wound site by secreting exosomes and regulated the polarization of macrophages. In another study, microarray experiments detected miRNA in exosomes derived from human adipose-derived mesenchymal stem cells (AEXOs). The results showed that the content of anti-inflammatory miRNAs, such as miR-223, miR-203, and miR-146a, was relatively high, and the researchers speculated that the anti-inflammatory effect of AEXO was attributed to these miRNAs (107). Overall, miRNAs derived from exosomes change the phenotype of macrophages and promote the resolution of inflammation to facilitate wound healing.

## 6 miRNA-Based Therapies and Future Perspective

It has been demonstrated that the expression of many miRNAs is dysregulated during wound healing, especially in chronic wounds caused by diabetes and a series of related complications (108). Given the critical role of miRNA in wound healing, it is considered an attractive candidate for a set of advanced treatment strategies. In addition, the application of miRNA in disease treatment has attracted great attention for the following reasons. A single miRNA can target multiple genes at the same time to form a network amplification effect. miRNA is a small molecule and relatively stable, which is conducive to technical manipulation.

The current miRNA-based treatment involves increasing or decreasing the expression level of a specific miRNA, which can be achieved through several technical methods. Increasing the expression of beneficial miRNAs is accomplished through the use of miRNA mimics, while methods to downregulate specific miRNAs include miRNA inhibitors, siRNA, and antisense oligonucleotides (109). However, naked miRNAs are not stable *in vivo* due to the action of nucleases. Furthermore, the ability of miRNAs to passively diffuse into target cells to exert regulatory effects is limited due to hydrophilic properties and their negative charges. Therefore, the successful delivery of miRNA mimics or anti-miR to the intended target also requires a safe and effective delivery system to reduce the degradation of the cargo in the body and maintain stability. Initially, viral vectors were recognized for efficiently carrying genetic material into cells and inducing stable expression. Unfortunately, these vectors often have the disadvantages of potential mutagenesis, toxicity and difficulty in production (109). In addition to traditional viral vectors, various non-viral vectors such as liposomes, nanoparticles and some inorganic materials, which have lower toxicity and higher efficiency, have also been developed (110). For example, Saleh B et al. used nanoparticle-laden hydrogels as a vector to deliver miR-223 to the wound site, which successfully weakened the inflammatory response and accelerated wound healing (54). A recent study reported that anti-inflammatory miR-146a could be effectively delivered to wound by nano silk solution to promote wound closure in diabetic mice (111). Moreover, miRNA as a cargo that presents in exosomes and participates in the regulation of the wound healing process also provides a new perspective for wound treatment.

According to statistics, more than 60 miRNA-based therapeutic drugs are in different stages of clinical trials, some of which have completed clinical trials and have been approved (112). Although miRNA-based treatment methods have shown promising results *in vivo*, they are still in preliminary stages for their applications to wound healing. It is a considerable challenge to modulate a process controlled by a complex and large network of interacting factors through a single miRNA. Therefore, a better understanding of the processes and functions of miRNA in wound healing is needed. Indeed, the selection of suitable target cells and an effective delivery system are also urgent problems to be solved in the future.

## 7 Conclusion

Inflammation is an effective defense mechanism to eliminate harmful damage, pathogen infection, and damaged cells at the wound site. Uncontrolled inflammation, however, can lead to delayed healing and eventually to chronic wounds. Therefore, the concept of how to adjust the balance between anti-inflammatory and pro-inflammatory activities at the chronic wound site and how to promote tissue repair have become a major challenge for physicians and many scientists.

Further exploration of the important role miRNA plays in wound inflammation will help us better understand normal and pathological wound healing mechanisms. While not without limitations, the current development of molecular-based therapies and new material carriers offers unprecedented opportunities for more effective wound management.

## Author Contributions

YJ and XX searched the literature and prepared the manuscript. YJ and LX organized the figure and table. LW and SQ examined and revised the manuscript. All authors contributed to the article and approved the submitted version.

## Funding

We greatly acknowledge the financial support from the National Nature Science Foundation of China (82074473, and 82104892), the Natural Science Foundation of Jiangsu province (BK20180001, BK20191201 and BE2020666), Jiangsu Province “333 Project” research project (BRA2020129), the Elderly Health Research Project of Jiangsu Province (LK2021043, and LR2021024).

## Conflict of Interest

The authors declare that the research was conducted in the absence of any commercial or financial relationships that could be construed as a potential conflict of interest.

## Publisher’s Note

All claims expressed in this article are solely those of the authors and do not necessarily represent those of their affiliated organizations, or those of the publisher, the editors and the reviewers. Any product that may be evaluated in this article, or claim that may be made by its manufacturer, is not guaranteed or endorsed by the publisher.
